# Unraveling the potential mechanisms of Xiaochaihu decoction alleviates metabolic associated fatty liver disease (MAFLD) by integrated transcriptomics, metabolomics, and network pharmacology

**DOI:** 10.1186/s13020-025-01310-y

**Published:** 2026-01-20

**Authors:** Ying Zhong, Xiao-min Zhu, Jian-chao Li, Li-song Sheng, Jia-ying Qi, Xian-hui Shen, Hang Du, Na-na Huang, Rong Sun

**Affiliations:** 1 The Second Qilu Hospital of Shandong University, Shandong University, 247 Beiyuan Ave, Jinan, 250033 Shandong China; 2 Shandong University of Traditional Chinese Medicine, 4655 Daxue Road, Jinan, 250355 Shandong China; 3 Advanced Medical Research Institute, Shandong University, 44 Wenhua Xi Road, Jinan, 250012 Shandong China; 4 Tianjin University of Traditional Chinese Medicine, 10 Poyanghu Road, Tianjin, 301617 China

**Keywords:** Xiaochaihu decoction, Metabolic associated fatty liver disease, Lipid droplets, Perilipins, Multi-omics

## Abstract

**Background:**

Metabolic associated fatty liver disease (MAFLD) has currently emerged as the predominant form of chronic liver diseases nowadays, with a high morbidity. Nevertheless, the general remission rate of current treatments for MAFLD remains unsatisfactory. The traditional Chinese medicine formulation Xiaochaihu decoction (XCHD) alleviates triglyceride (TG) and total cholesterol (TC) in patients with fatty liver disease, but the precise mechanisms remain to be determined.

**Purpose:**

This study aimed to investigate the effects and underlying mechanisms of XCHD on MAFLD.

**Methods:**

The components of XCHD and XCHD-containing serum were characterized by UPLC/MS. High-fat diet and glucose-fructose water-induced MAFLD model in mice was established to evaluate the effects of XCHD. Non-targeted metabolomics, RNA-sequencing, and Network pharmacology were performed and integrated in the mice liver. Molecular biology experiments, like Western blot, were taken to investigate its potential mechanisms. Finally, the effects of PPARγ, perilipin 2 (PLIN2), and perilipin 3 (PLIN3) were detected by over-expressed PPARγ or suppressed PPARγ.

**Results:**

XCHD markedly alleviates MAFLD by reducing lipid droplets, TC, and TG accumulation in MAFLD mice and free fatty acid induced HepG2 and AML12 cells. Non-targeted metabolomics suggested that XCHD reduced hepatic lysophospholipid, and RNA-sequencing revealed that the underlying mechanism would be related to lipid droplet formation. Molecular biology experiments indicated that XCHD decreased PLIN2 and PLIN3 in vivo and in vitro. Network pharmacology analysis revealed that the mechanism of XCHD against MAFLD involves the PPARγ signaling pathway. Molecular biology experiments indicated that XCHD decreased PPARγ in vivo and in vitro. Overexpression of PPARγ indicated that XCHD exert regulatory effects through PPARγ-mediated inhibition of PLIN2 and PLIN3. However, the inhibitory effects of XCHD on PLIN2 and PLIN3 are not solely dependent on this pathway.

**Conclusion:**

XCHD alleviates MAFLD by decreasing lysophospholipid, regulating liver lipid droplets formation, and targeting PLIN2/PLIN3. PPARγ was one of the cross in for PLIN2/PLIN3 pathway, which provides novel insights for MAFLD therapy.

**Supplementary Information:**

The online version contains supplementary material available at 10.1186/s13020-025-01310-y.

## Introduction

Metabolic-associated fatty liver disease (MAFLD), previously known as non-alcoholic fatty liver disease (NAFLD), is characterized by excessive accumulation of hepatic lipids [1,2]. As the most prevalent chronic liver disease in the twenty-first century, affecting nearly 30% of the adult population and 80% of obese individuals [3], the prevalence of MAFLD is steadily increasing. MAFLD is characterized by differential progression rates and clinical outcomes, presumably driven by distinct pathogenesis from simple steatosis, to metabolism-associated steatohepatitis (MASH), liver inflammation, fibrosis, cirrhosis, and hepatocellular carcinoma [4]. Although Rezdiffra [5], a thyroid hormone receptor agonist, has been approved by the FDA for MASH, and other drugs like glucagon-like peptide-1 (GLP-1) agonists, steroidal farnesoid X nuclear receptor (FXR) ligands show potential in large randomized trials, high prevalence of MAFLD coupled with obesity, and type 2 diabetes mellitus will persistently impose substantial physical, psychological, and economic burdens on affected individuals [6,7]. Consequently, there remains an urgent need for novel therapeutics for MAFLD.

In the course of MAFLD pathogenic drivers discovery, a theory was recognised that a large number of toxic lipid species accumulate and lead to hepatocellular stress, injury, and death because of metabolic disturbances in the liver [8]. Lipid droplets (LDs) are dynamic and energetically active lipid storage vesicles within eukaryotic cells, formed by a core of neutral lipids and surrounded by a monolayer phospholipid membrane containing phospholipids [9], structural proteins, functional enzymes, and membrane transport proteins, which perform pivotal functions in lipid metabolism, energy balance, and signal transduction [10]. Hepatic LD accumulation is an adaptive response to increased free fatty acid (FFAs) from diet, adipose tissue, and synthesis in hepatocytes. When FFAs accumulate, transcriptional factors like sinsulin responsive sterol regulatory element binding protein1 (SREBP1) active, lipogenic gene expression increases, FFAs are transported into the endoplasmic reticulum to be converted into neutral lipids, ultimately stored as LDs or secreted as very low-density lipoproteins (VLDL) [11,12]. LDs have been demonstrated to modulate the release and utilisation of FFAs through lipolysis and lipophagocytosis, thus ensuring homeostasis. It is important to note that excessive FFAs trigger endoplasmic reticulum stress and oxidative stress, thereby establishing a vicious circle that exacerbates LDs accumulation. A substantial body of evidence has emerged that demonstrates the accumulation of LDs in excess of physiological levels, in conjunction with a dysregulation of the processes governing their biogenesis and subsequent degradation, represents the primary initiating steps in the pathogenesis of MAFLD. This dysregulation activates the pathogenesis mechanism through enhanced intracellular lipid buffering, consequently leading to steatosis, hepatocyte inflammation, and fibrosis. [13,14].

Traditional Chinese medicine (TCM) has been proven to be reliable and effective by numerous clinical trials. Xiaochaihu decoction (XCHD), consists of Bupleuri Radix (Chaihu in Chinese, CH), Scutellariae Radix (Huangqin in Chinese, HQ), Ginseng Radix et Rhizoma (Renshen in Chinese, RS), glycyrrhizae Radix et Rhizoma (Gancao in Chinese, GC), Pinelliae Rhizoma (Banxia in Chinese, BX), Rhizoma Zingiberis Recens (Shengjiang in Chinese, SJ), and Jujubae Fructus (Dazao in Chinese, DZ), was a classic TCM formula for liver diseases because of its pattern of “reconciling shaoyang”. XCHD was first recorded in *Shang Han Lun*, written by Zhang Zhongjing in the Han Dynasty. As modern medicine advanced, XCHD was regarded as an efficient prescription for cold [15], steatohepatitis [16], gastroesophageal reflux disease [17], depression, and melancholia [18] in the clinic. Network pharmacology reveals that the core functions of XCHD on MAFLD were immunity regulation, metabolism regulation, and oxidative stress regulation [19]. It indicates that components extracted from CH, HQ, RS, and GC have an ameliorative effect on MAFLD. In addition, our studies had suggested that saikosaponin A and saikosaponin D, main bioactive compounds in the core herb CH of XCHD, indicated effects of preventing glycerolipid accumulation, inhibiting FFAs biosynthesis, promoting FFAs degradation, and recovering hepatic lipid homeostasis in NAFLD model mice [20,21]. However, the underlying mechanism of XCHD in treating MAFLD remains largely unclear.

In this study, we evaluate the effects of XCHD on high-fat diet and glucose-fructose water-induced MAFLD in mice and the potential operating mechanisms. We performed transcriptomics, metabolomics, Network pharmacology, and experiments on mouse livers or liver cell lines to prove that XCHD regulates lipid metabolism through PPARγ/PLIN2/PLIN3. Collectively, our results indicate that XCHD may serve as a potential therapeutic option in the treatment of MAFLD.

## Materials and methods

### Materials

CH (Lot No. 210601), HQ (Lot No. 200501), BX (Lot No. 210701), RS (Lot No. 200701), GC (Lot No. 150501), DZ (Lot No. 230101) were purchased from Shandong Baiweitang Chinese Herbal Medicine Drinks Slice Co., Ltd (China). Rosiglitazone (R2408) was purchased from Sigma (St. Louis, MO, USA). T0070907 (HY-13202) was purchased from MCE. HRP conjugated goat anti-rabbit IgG (H + L) was purchased from Servicebio (Wuhan, China). First antibodies used for western blot analysis targeting PLIN2 (ab108323) were purchased from Abcam (Cambridge, MA, USA). Antibodies PPARγ (2435S) were purchased from Cell Signaling Technology Inc. (Danvers, MA, USA). Antibodies Plin3 (10694–1-AP), Plin5 (26951–1-AP), and β-actin (81115-1-RR) were purchased from Proteintech Group (Rosemont, IL, USA). Mito-Tracker Red CMXRos (C1049B), saponin (P0095), and BODIPY 493/503 (C2053S) were purchased from Beyotime (Shanghai, China). ALT, AST, TC, TG, LDL-c, and NAFA assay kits were purchased from Nanjing Jiancheng Bioengineering Institute (Nanjing, China).

### Preparation of XCHD

According to Shan Han Lun, the dose of XCHD was 78 g of crude herbs, including 24 g CH, 9 g HQ, 9 g BX, 9 g RS, 9 g GC, 9 g SJ, and 4 DZ (9 g). Besides SJ, all the herbs that consisted of XCHD were passed quality control administered by Prof. Qingmei Guo (Shandong University of Traditional Chinese Medicine). The human dose was converted to the mouse dose based on the body surface area with a coefficient of 9.1 as 10.14 g/kg/day (low dose). The medium dose was 20.28 g/kg/day, and the high dose was 40.56 g/kg/day. Herbs were precisely weighed according to the original formula in Shan Han Lun, immersed in tenfold volumes of distilled water for 30 min, and produced as a decoction by the condensation reflux method for 1.5 h two times. Two extractions were mixed and concentrated to 4.056 g crude drug/mL in a rotary evaporator. Part of the decoction was diluted to 50 mg crude drug/mL in distilled water and filtered through a 0.22 μm membrane filter for UHPLC-MS/MS and cell experiments.

### Preparation of rat XCHD-loaded serum

Male SD rats, weighing 180–200 g, were purchased from Jinan 220 Pengyue Experimental Animal Breeding Co., Ltd. The rat equivalent dose was calculated based on a double daily clinical human dose by the coefficient of 6.3. Rats were divided into two groups: CON (administered with physiological saline) and XCHD-M (administered with 7.02 g/kg XCHD). Rats were treated for 3 days, and on the last day, 0.5 h after treatment, blood samples were collected from the heart. Following a two-hour resting period at room temperature, the blood sample was placed in a centrifuge at 3000 rpm for 15 min. The upper layer was then stored in a −20 °C environment.

### UHPLC-MS/MS for the identification

The whole Ultra-high-performance liquid chromatography-mass spectrometry/mass spectrometry (UHPLC-MS/MS) procedure is implemented on UPLC-Q-Exactive Orbitrap-MS. Male C57BL/6 J mice were treated with 0.4056 g/mL XCHD (0.1 ml of solution per 10 g body weight by oral gavage) for 2 h. Mouse serum was taken and prepared as serum samples for UHPLC-MS/MS. The separations of XCHD were performed on an Agilent Poroshell 120 SB-C18 column (2.1 mm × 100 mm, 1.9 μm) at 35 ◦C with a flow rate set at 0.3 mL/min. Acetonitrile (A) and 0.1% formic acid solution (B) were used as mobile phases. The gradient elution program was performed as shown: 95% B at 0–2 min, 95–60% B at 2–8 min, 60–40% B at 8–14 min, 40–20% B at 14–21 min, 20–5% B at 21–27 min, 95–5% B at 27–30 min. The final supernatant was analyzed using a Waters H-Class UPLC system coupled to an AB Sciex Triple TOF® 4600 mass spectrometer. The UHPLC effluents were ionized by employing a heated electrospray ionization (HESI) source in ESI + or ESI- mode. The exact conditions for mass spectrometry are as follows: spray voltage (ESI +) of 3.5 kV, spray voltage (ESI-) of 2.9 kV, capillary temperature of 320  C, and the range of m/z (mass-to-charge ratio) of 100–1500 under full-scan data.

### Animal study

Male C57BL/6 J mice (18–20 g) were purchased from Vital River Laboratory Animal Technology Co., Ltd (Beijing, China) (SCXK(Jing)2021-0006) in a barrier system with a 12 h/12 h light and dark cycle, accompanied by a regulated temperature (22 ± 1  C). Mice were supplied with sterilized water and normal chow for a week of acclimation. Thereafter, the mice were divided into 6 groups randomly: (1) NOR group fed with normal chow and sterilized water; (2–5) MOD, XCHD-L, XCHD-M, XCHD-H, and YSF groups fed with HFSW, which contained a western diet (42% kcal from fat and 0.2% cholesterol) and a high-fructose-glucose solution (18.9 g/L D-glucose and 23.1 g/L D-fructose) (HFSW) [22]. Four weeks later, mice were treated with sterile water for NOR and MOD groups, 0.1014 g/ml, 0.2028 g/ml, 0.4056 g/ml XCHD for XCHD-L, M, and H groups, and 0.1778 g/ml polyene phosphatidylcholine for the YSF group as a positive control. (0.1 ml of solution per 10 g body weight by oral gavage). The duration of oral gavage lasted for four weeks, in parallel with the continued administration of HFSW. All animal experiments were approved by the Research Ethics Committee of the Second Hospital of Shandong University (KYLL2024910).

### Non-targeted metabolomics analysis

Mouse liver tissues from NOR, MOD, and XCHD-M groups were used for non-targeted metabolomics based on liquid–liquid-mass spectrometry (LC–MS) technology by Novogene Co., Ltd. (Beijing, China). The raw files (.raw) obtained by mass spectrometry were imported into Compound Discoverer 3.3 (CD3.3) to obtain the qualitative and quantitative results of the metabolites by matching the identification of molecular peaks with high-quality mzCloud databases built from standards with mzVault and MassList databases. Analysis of differential metabolites was performed by the edgeR R package (3.18.1) with a corrected criterion of VIP > 1.0, FC > 1.5 or FC < 0.667, and P-value < 0.05 as the threshold for significance. Online metabolic databases, including Lipidmaps (https://lipidmaps.org), HMDB (https://hmdb.ca/), and PubChem (http://pubchem.Ncbi.nlm.nih.gov), with exact masses of the metabolites, were used to identify and categorize the differential metabolites.

### RNA-sequencing and bioinformatic analysis

Total liver RNA in 3 mice for NOR, MOD, and XCHD-M groups were selected for RNA-sequencing analysis by Novogene Co., Ltd. (Beijing, China). RNA-sequencing was performed by Agilent 2100 bioanalyzer for quality control and NEB method for library establishment. The initial quantification was performed by Qubit2.0 Fluorometer, and the library insert size was detected by Agilent 2100 Bioanalyzer. Once the insert size was as anticipated, qRT-PCR was performed to quantify the effective concentration of the library, which should be higher than 1.5 nM to ensure its quality. Following the library testing, the different libraries were pooled and sequenced by Illumina. Analysis of differential expression was performed by the edgeR R package (3.18.1). Genes expression |log2FoldChange|> 1 and p-value < 0.05 were regarded as differentially expressed genes (DEGs). Gene ontology (GO) enrichment analysis, Kyoto Encyclopedia of Genes and Genomes (KEGG) analysis, and Reactome analysis of differentially expressed genes were implemented by Metascape (https://www.metascape.org). DEGs were also analyzed based on the GO dataset using a local version of the Gene Set Enrichment Analysis (GSEA) tool. Correlation analysis of transcriptome and metabolome was performed by Pearson correlation analysis. Pearson correlation analysis was performed to analyze the relationship between transcriptome and metabolome (|r|> 0.8 and p < 0.05 were regarded as significant).

### Network pharmacology

Effective compounds of CH, HQ, RS, GC, BX, SJ, and DZ were retrieved from TCMSP (https://old.tcmsp-e.com/tcmsp.php), which were selected as oral bioavailability ≥ 30 and drug similarity ≤ 0.18, or UHPLC-MS/MS. Targets of each effective compound were obtained from TCMSP and PubMed. And targets of NAFLD were found in CTD (https://ctdbase.org/). All the targets were merged, and repeated targets were selected to perform a protein–protein interaction network through STRING (https://cn.string-db.org/) and Cytoscape v3.7.2. Finally, all the selected targets were put into the Metascape database (https://www.metascape.org) for KEGG and GO enrichment analysis.

### Cell culture and in vitro study

Human liver cancer cell line (HepG2 cells) and mouse liver cell line (AML12 cells) were obtained from Shanghai Institutes for Biological Sciences and maintained at 37 ℃ in a humidified atmosphere containing 5% CO_2_. Cells (1 × 10^4^ cells/a well in 96-well culture plates and 2 × 10^5^ cells/a well in 6-well culture plates) were seeded in culture plates for 24 h. Excluding the NOR group, 500 μM OAPA solution (Oleic acid: palmitic acid = 2:1) was administered for 24 h to promote lipogenesis, and various concentrations of XCHD (0, 5%, 10%, 20%) were added. After 24 incubation, HepG2 or AML12 cells were collected for oil red O staining (servicebio, Wuhan, China), BODIPY 493/503 staining (Beyotime, Shanghai, China), and western blot experiments.

### BODIPY 493/503 staining

HepG2 cells and AML12 cells were maintained at 37 ℃ in a humidified atmosphere containing 5% CO_2_. Cells (1 × 10^4^ cells/well in 96-well black culture plates) were seeded in culture plates for 24 h. Excluding the NOR group, 500 μM OAPA solution (Oleic acid: palmitic acid = 2:1) was administered for 24 h to promote lipogenesis, and various concentrations of XCHD (0, 5%, 10%, 20%) were added. After 24 h incubation, cells were washed with PBS for 2 times and co-incubated with BODIPY 493/503 and Hoechst 33342 mixed solution for 15 min. After 2 times washing with PBS, plates were put into Cytation 5 Cell Imaging Multi-Mode Reader (Biotek Cytation5) and detected at 475/516 and 350/461. Then, images were taken by an Inverted fluorescence microscope (Olympus IX73).

### Immunofluorescence

AML12 cells were maintained at 37 ℃ in a humidified atmosphere containing 5% CO_2_. Cells (5 × 10^4^ cells/a well Confocal Dish) were seeded in culture plates for 24 h. Excluding the NOR group, 500 μM OAPA solution (Oleic acid: palmitic acid = 2:1) was administered for 24 h to promote lipogenesis, and XCHD-M were treated for 24 h. Cells were incubated with Mito-Tracker Red CMXRos or BODIPY 493/503 for 20 min at 37  C. After that, cells were fixed with 4% paraformaldehyde for 20 min, permeabilized with saponin, and blocked with 5% goat serum. Subsequently, cells were incubated with primary antibodies (PLIN2 or PLIN3) for 16 h, followed by fluorescently labeled secondary antibodies for 1 h. After that, the nuclei were stained by DAPI for 1 min, and fluorescent images were acquired by Leica MICA.

### Overexpression of PPARγ

HepG2 cells and AML12 cells were maintained at 37 ℃ in a humidified atmosphere containing 5% CO_2_. Cells (2 × 10^5^ cells/well in 6-well culture plates) were seeded in culture plates for 24 h. Excluding the NOR group, 500 μM OAPA solution (Oleic acid: palmitic acid = 2:1) was administered for 24 h to promote lipogenesis, and XCHD-M, Rosig (10 nM), and XCHD-M plus Rosig were treated. After 24 incubation, HepG2 or AML12 cells were collected for western blot experiments. AML12 Cells (2 × 10^5^ cells/well in 6-well culture plates) were seeded in culture plates for 24 h. 3ug per well mouse Pparg vector (NM_011146, pcDNA3.1-3xFlag-C) were added in PPAR + groups, while 3ug per well control vector were added in NC group. After 4 h incubation, the medium with vectors was replaced by complete medium with 500 μM OAPA. After 24 h of incubation, cells were administered by XCHD-M for 24 h. Then, cells were collected for Western blot experiments.

### Statistical analysis

All data are presented as mean ± standard deviation(SD), performed with the GraphPad Prism 8 software package (GraphPad, La Jolla, CA, USA). Statistical analysis was performed using Student’s t-test among multiple groups using at least three independent experiments. P-value ≤ 0.05 was considered statistically significant.

## Results

### Chemical characterization of XCHD

**Fig.1:**
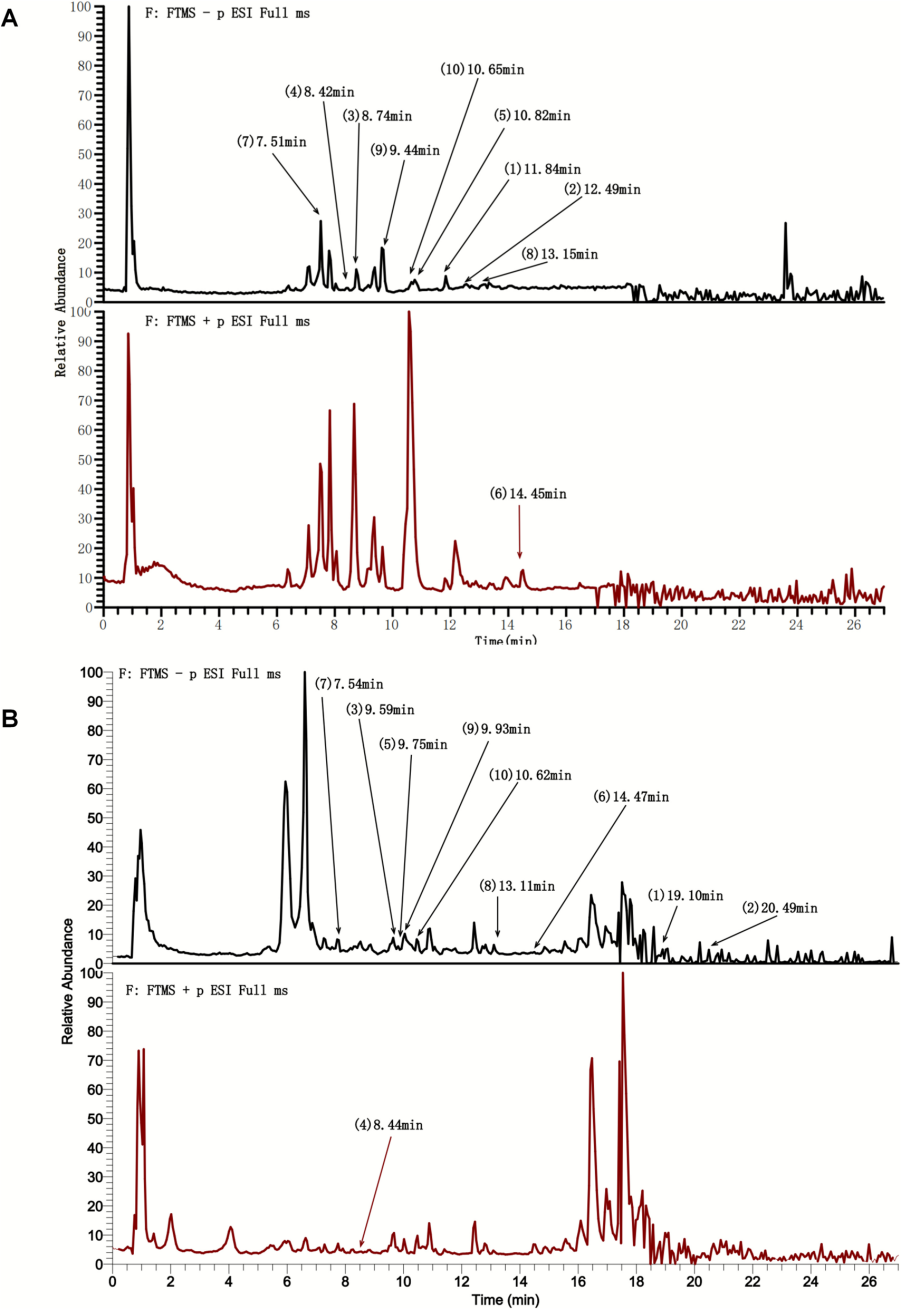
Chemical characterization of XCHD. The total ion current chromatograms (TICCs) of the prepared XCHD and serum samples are shown in **A** and **B** with positive (ESI +) ionization modes in red and negative (ESI−) ionization modes in black

UHPLC-MS/MS was used to identify the XCHD. 42 best match and 56 best match compounds were obtained from XCHD, and XCHD contained serum (Table 1 and Table 2). The representative compounds of each herb in XCHD were retrieved, including saikosaponin A (1), saikosaponin D (2), baicalin (3), ginsenoside Rg1 (4), ginsenoside Re (5), beta-sitosterol (6), liquiritin (7), 6-gingerol (8), quercetin (9), and vanillic acid 4-β-D-glucopyranoside (10). The peak and departure times of each component were given in Fig. 1(A) for XCHD aqueous extracts and Fig. 1(B) for serum samples. Detailed mass spectrum images of each component were provided in Fig. S1A-J.

**Table 1:** The best match chemical compositions of XCHD

NO	RT (min)	Name	Formula	Neutral mass (Da)	Observed (m/z)	Error (ppm)	Adduct
1	0.87	α,α-Trehalose	C12 H22 O11	342.11597	341.10857	−0.72	[M-H]−1
2	0.86	Calystegine A7	C7 H13 N O3	159.08876	160.09603	−4.95	[M + H] + 1
3	0.87	D-(+)-Proline	C5 H9 N O2	115.06303	116.07031	−2.62	[M + H] + 1
4	0.75	Choline	C5 H13 N O	103.09962	104.10689	−0.93	[M + H] + 1
5	0.86	5-({[3-chloro-5-(trifluoromethyl)−2-pyridyl]methyl}thio)−4-pentyl-4H-1,2,4-triazol-3-ol	C14 H16 Cl F3 N4 O S	380.06998	381.07726	3.78	[M + H] + 1
6	0.86	Caprolactam	C6 H11 N O	113.08381	114.09109	−2.23	[M + H] + 1
7	0.84	D-(-)-Fructose	C6 H12 O6	180.06246	179.05506	−5.16	[M-H]−1
8	0.86	Betaine	C5 H11 N O2	117.07863	118.08593	−2.97	[M + H] + 1
9	0.90	1-(Mesitylsulfonyl)−2,4-dimethyl-1H-pyrrole	C15 H19 N O2 S	277.11478	278.12205	4.07	[M + H] + 1
10	0.86	Trigonelline	C7 H7 N O2	137.04704	138.05431	−4.69	[M + H] + 1
11	0.85	D-(+)-Arabitol	C5 H12 O5	152.06733	151.05998	−7.52	[M-H]−1
12	0.87	morpholine-4-carboximidamide hydrobromide	C5 H11 N3 O	129.08974	130.09699	−3.65	[M + H] + 1
13	1.04	Adenosine	C10 H13 N5 O4	267.09559	268.10286	−4.37	[M + H] + 1
14	1.05	4-Oxoproline	C5 H7 N O3	129.04134	128.03406	−9.73	[M-H]−1
15	0.86	Adenine	C5 H5 N5	135.05388	136.06117	−4.52	[M + H] + 1
16	1.07	Dibutylamine	C8 H19 N	129.15137	130.15865	−2.91	[M + H] + 1
17	1.01	NP-021733	C7 H13 N O2	143.09399	144.10126	−4.47	[M + H] + 1
18	1.04	L-Pyroglutamic acid	C5 H7 N O3	129.04218	130.04945	−3.23	[M + H] + 1
19	0.83	N,N′-Bis(2-methoxyethyl)−4,6-dinitro-1,3-benzenediamine	C12 H18 N4 O6	314.12116	313.11391	−4.68	[M-H]−1
20	1.05	diselenide	H2 Se2	161.84822	160.84094	−2.95	[M-H]−1
21	0.95	Pipecolic acid	C6 H11 N O2	129.07849	130.08576	−3.82	[M + H] + 1
22	0.87	4-Amino-3-[(E)-{4′-[(E)-(2,4-diaminophenyl)diazenyl]−4-biphenylyl}diazenyl]−5-hydroxy-6-[(E)-phenyldiazenyl]−2,7-naphthalenedisulfonic acid	C34 H27 N9 O7 S2	737.14431	736.13703	−4.31	[M-H]−1
23	1.04	Phenylacetylene	C8 H6	102.04669	120.0805	−2.54	[M + NH4] + 1
24	0.84	3-(2-Fluoropropyl)thiophene	C7 H9 F S	144.04101	143.03374	0.74	[M-H]−1
25	6.07	PEG n7	C14 H30 O8	326.19235	344.22619	−5.26	[M + NH4] + 1
26	1.03	Nicotinic acid	C6 H5 N O2	123.03171	124.03901	−2.61	[M + H] + 1
27	1.01	Nicotinamide	C6 H6 N2 O	122.04771	123.05499	−2.45	[M + H] + 1
28	0.87	5-Ketofructose	C6 H10 O6	178.04718	415.10892	−3.11	[2 M-H + HAc]−1
29	1.05	2-C-methyl-D-erythritol-4-phosphate	C5 H13 O7 P	216.0393	215.03202	−2.72	[M-H]−1
30	0.90	7-Amino-1H-imidazo[4′,5′:4,5]thieno[3,2-d]pyrimidin-5(8H)-one	C7 H5 N5 O S	207.02192	208.02915	2.15	[M + H] + 1
31	1.04	Uridine	C9 H12 N2 O6	244.06905	243.06177	−2	[M-H]−1
32	1.49	5-Carboxybenzofuroxan	C7 H4 N2 O4	180.01789	181.02525	4.36	[M + H] + 1
33	0.96	4-(Boc-amino)−1-butanol	C9 H19 N O3	189.13567	172.13237	−4.34	[M + H-H2O] + 1
34	7.51	Liquiritin	C21 H22 O9	418.11801	417.11887	2.06	[M-H]-
35	8.74	Baicalin	C21 H18 O11	446.07654	445.07751	2.18	[M-H]-
36	8.44	Ginsenoside Rg1	C42 H72 O14	423.36214	423.36002	5.01	[M-OGlu-Glu + 3H] +
37	9.46	Quercetin	C15 H10 O7	302.03427	301.03537	3.65	[M-H]-
38	10.82	Ginsenoside Re	C48 H82 O18	946.54174	945.54108	0.70	[M-H]-
39	11.84	Saikosaponin A	C42 H68 O13	780.45672	779.45789	1.50	[M-H]-
40	12.49	Saikosaponin D	C42 H68 O13	780.45672	779.4577	1.26	[M-H]-
41	13.15	[6]-Gingerol	C17 H26 O4	294.17474	293.1759	3.96	[M-H]-
42	10.62	Vanillic acid 4-β-D-glucopyranoside	C14 H18 O9	330.08671	329.23325	445.094777	[M-H]-

**Table 2:** The best match chemical compositions of XCHD contained serum

NO	RT (min)	Name	Formula	Neutral mass (Da)	Observed (m/z)	Error (ppm)	Adduct
1	0.94	11,12-Epoxy-(5Z,8Z,11Z)-icosatrienoic acid	C4 H9 N3 O2	131.06899	132.0762	−3.74	[M + H] + 1
2	23.53	3-Indoxyl sulphate	C22 H32 O2	328.23988	327.2326	−1.07	[M-H]−1
3	0.94	3-Indoxyl sulphate	C5 H9 N O2	115.06303	116.0703	−2.63	[M + H] + 1
4	1.06	9(Z),11(E)-Conjugated linoleic acid	C6 H8 O9 S	255.98846	254.98118	−1.74	[M-H]−1
5	9.66	3-Indoxyl sulphate	C9 H7 N	129.05732	130.0646	−4.07	[M + H] + 1
6	24.06	Creatine	C20 H32 O2	304.23974	303.23263	−1.63	[M-H]−1
7	11.09	Docosahexaenoic acid	C24 H40 O5	408.28749	453.28573	−0.21	[M + FA-H]−1
8	19.10	D-(+)-Proline	C30 H46 O4	470.33728	471.34456	−4.95	[M + H] + 1
9	1.677	L-Ascorbic acid 2-sulfate	C11 H12 N2 O2	204.08895	205.09623	−4.52	[M + H] + 1
10	6.074	Quinoline	C8 H7 N	117.05756	118.06484	−2.44	[M + H] + 1
11	1.00	Arachidonic acid	C6 H11 N O2	129.07846	130.08574	−4	[M + H] + 1
12	0.94	β-Muricholic acid	C7 H7 N O2	137.047	138.05428	−4.99	[M + H] + 1
13	4.06	18-β-Glycyrrhetinic acid	C11 H12 N2 O2	204.0889	203.08173	−4.81	[M-H]−1
14	11.40	DL-Tryptophan	C24 H40 O5	408.28755	453.28585	−0.05	[M + FA-H]−1
15	19.47	Indole	C30 H46 O4	470.33827	469.3319	−2.84	[M-H]−1
16	7.74	L(-)-Pipecolinic acid	C9 H7 N	129.05737	130.06465	−3.69	[M + H] + 1
17	2.74	Trigonelline	C9 H17 N O5	219.1097	220.11683	−4.45	[M + H] + 1
18	7.54	DL-Tryptophan	C8 H7 N O4 S	213.00879	212.00151	−3.71	[M-H]−1
19	14.47	β-Muricholic acid	C13 H10 O	182.07229	183.07956	−4.83	[M + H] + 1
20	10.47	18-β-Glycyrrhetinic acid	C12 H21 N	179.16657	180.17385	−4.62	[M + H] + 1
21	14.50	Quinoline	C9 H10 O	134.07254	135.07982	−4.64	[M + H] + 1
22	5.77	Pantothenic acid	C12 H12 N2 O2	216.08883	217.0961	−4.86	[M + H] + 1
23	6.97	3-Indoxyl sulphate	C13 H25 N O4	259.17712	260.1844	−4.76	[M + H] + 1
24	1.08	Benzophenone	C5 H7 N O3	129.04207	130.04934	−4.09	[M + H] + 1
25	4.07	Rimantadine	C9 H7 N	129.05729	130.06457	−4.34	[M + H] + 1
26	19.84	2,4-Dimethylbenzaldehyde	C20 H32 O3	320.23497	319.2277	−0.54	[M-H]−1
27	11.41	2,3,4,9-Tetrahydro-1H-β-carboline-3-carboxylic acid	C10 H12 O2	164.08297	165.09025	−4.61	[M + H] + 1
28	8.66	Hexanoylcarnitine	C9 H7 N	129.05738	130.06465	−3.65	[M + H] + 1
29	25.37	L-Pyroglutamic acid	C20 H34 O2	306.2557	305.24842	−0.59	[M-H]−1
30	6.85	Quinoline	C9 H11 N O2	165.07828	166.08552	−4.24	[M + H] + 1
31	8.66	20-Hydroxy-(5Z,8Z,11Z,14Z)-eicosatetraenoic acid	C10 H9 N O2	175.06259	176.06986	−4.24	[M + H] + 1
32	16.43	4-Phenylbutyric acid	C18 H32 O2	280.23991	279.23264	−1.13	[M-H]−1
33	22.95	Quinoline	C16 H32 O3	272.23514	271.22787	0	[M-H]−1
34	18.78	8Z,11Z,14Z-Eicosatrienoic acid	C14 H26 O4	258.18188	259.18924	−4.76	[M + H] + 1
35	10.86	L-Phenylalanine	C24 H40 O5	408.28749	407.28008	−0.22	[M-H]−1
36	22.26	2-(1H-indol-3-yl)acetic acid	C20 H30 O2	302.22431	301.21703	−0.89	[M-H]−1
37	7.86	Linoleic acid	C11 H11 N O3	205.07294	206.08024	−4.63	[M + H] + 1
38	16.75	16-Hydroxyhexadecanoic acid	C16 H32 O3	272.23502	271.22774	−0.47	[M-H]−1
39	7.87	Dibutyl hexanedioate	C13 H14 N2 O3	246.10005	245.09277	−1.6	[M-H]−1
40	11.52	β-Muricholic acid	C12 H18 O3	210.12458	211.13182	−4.82	[M + H] + 1
41	11.91	Eicosapentaenoic acid	C16 H12 O7	316.0577	315.05091	−1.91	[M-H]−1
42	14.81	Indole-3-lactic acid	C18 H34 O4	314.24555	313.23827	−0.51	[M-H]−1
43	11.92	16-Hydroxyhexadecanoic acid	C15 H10 O6	286.04759	285.04031	−0.53	[M-H]−1
44	9.74	N-Acetyl-DL-tryptophan	C12 H22 O4	230.1512	229.14393	−2.63	[M-H]−1
45	15.16	Jasmonic acid	C11 H14 O3	194.09339	195.10067	−4.65	[M + H] + 1
46	0.94	3-Methoxy-5,7,3',4'-tetrahydroxy-flavone	C4 H9 N3 O2	131.06899	132.0762	−3.74	[M + H] + 1
47	23.53	(±)9(10)-DiHOME	C22 H32 O2	328.23988	327.2326	−1.07	[M-H]−1
48	0.94	Luteolin	C5 H9 N O2	115.06303	116.0703	−2.63	[M + H] + 1
49	1.06	Dodecanedioic acid	C6 H8 O9 S	255.98846	254.98118	−1.74	[M-H]−1
50	9.66	4-Ethoxy ethylbenzoate	C9 H7 N	129.05732	130.0646	−4.07	[M + H] + 1
51	7.54	Liquiritin	C21 H22 O9	418.11801	417.11908	2.57	[M-H]-
52	8.44	Ginsenoside Rg1	C42 H72 O14	423.36214	423.36041	4.09	[M-OGlu-Glu + 3H] +
53	9.46	Quercetin	C15 H10 O7	302.03427	301.03537	3.65	[M-H]-
54	9.75	Ginsenoside Re	C48 H82 O18	946.54174	945.55756	16.7	[M-H]-
55	19.1	Saikosaponin A	C42 H68 O13	780.45672	469.33099	0.53	[M-Glu-Fuc-H]-
56	13.11	[6]-Gingerol	C17 H26 O4	294.17474	293.17575	3.45	[M-H]-

### XCHD alleviates HFSW-induced hepatic steatosis in mice

**Fig. 2:**
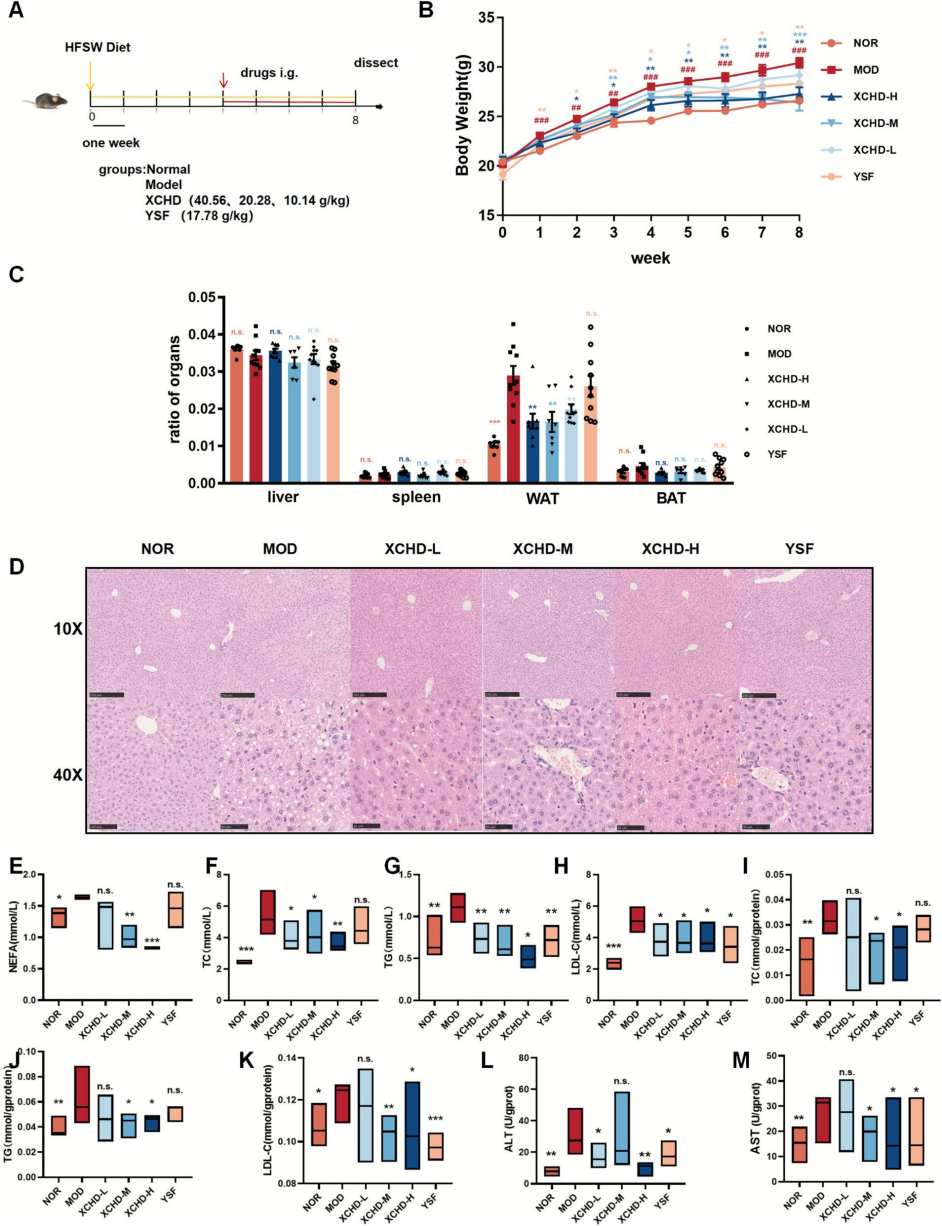
XCHD alleviates HFSW-induced hepatic steatosis in mice. **A** HFSW-induced MAFLD model method. **B** The weekly body weight of mice (*n* = 10). **C** organs/body weight of mice (*n* = 10). **D** Typical photos of H&E staining of liver tissues. **E** Levels of mice serum nonesterified fatty acid (*n *= 6). **F**–**H** Levels of mice serum TC, TG, and LDL-C (*n* = 6). **I**–**K** Levels of mice liver TC, TG, and LDL-C (*n* = 6). **L**–**M** Levels of mice serum ALT and AST (*n* = 6). Data are represented using at least three independent experiments as the mean ± SEM. *p < 0.05, **p < 0.01, vs. MOD group, ^#^p < 0.05, ^##^p < 0.01, ^###^p < 0.001 NOR vs. MOD group

To investigate the effects of XCHD on NAFLD, mice were administered XCHD (L, M, H) for 4 weeks under the HFSW-induced NAFLD model (Fig. 2A). The MOD group had markedly higher body weight compared with the NOR group. XCHD attenuated weight gain under HFSW-induced conditions (Fig. 2B, Fig. S2C), but food intake and water intake were not significantly different between groups (Fig. S2A-B). Detection of organs/body weight revealed that significant decrease in the weight of white adipose tissue (WAT) while non-significant alterations in liver, spleen, and brown adipose tissue (BAT) in the XCHD group compared with the MOD group (Fig. 2C). Livers in the MOD group were yellow, while XCHD group appeared reddish-brownto the naked eye (Fig. S2 D). Consistent with it, histopathological analysis of hepatic tissues revealed a notable decrease in hepatic steatosis, with reduced lipid droplet accumulation in a dose-dependent pattern (Fig. 2D). To validate the role of XCHD on liver metabolism, liver tissue and serum biochemistry were analyzed. The result indicated that XCHD markedly decreased triglycerides (TG), total cholesterol (TC), low-density lipoprotein cholesterol (LDL-C), and nonesterified fatty acid (NEFA) levels in mice serum (Fig. 2E–H). The variation of TG, TC, and LDL-C in the liver is consistent with serum (Fig. 2I–K). Treatment with the XCHD resulted in a marked decrease in fasting blood glucose levels and an enhanced insulin sensitivity, as evidenced by the insulin tolerance test (ITT) (Fig. S2E-F). In addition, the results also indicated that sensitive biomarkers of hepatocellular integrity, aminotransferase (ALT) and aspartate aminotransferase (AST) in mice serum, which provided compelling evidence that XCHD exerted a protective effect against hepatocellular injury in the MAFLD model mice (Fig. 2L, M). In brief, XCHD significantly inhibited the HFSW-caused extensive steatosis in mice.

### XCHD modulates HFSW-induced hepatic metabolism

**Fig. 3:**
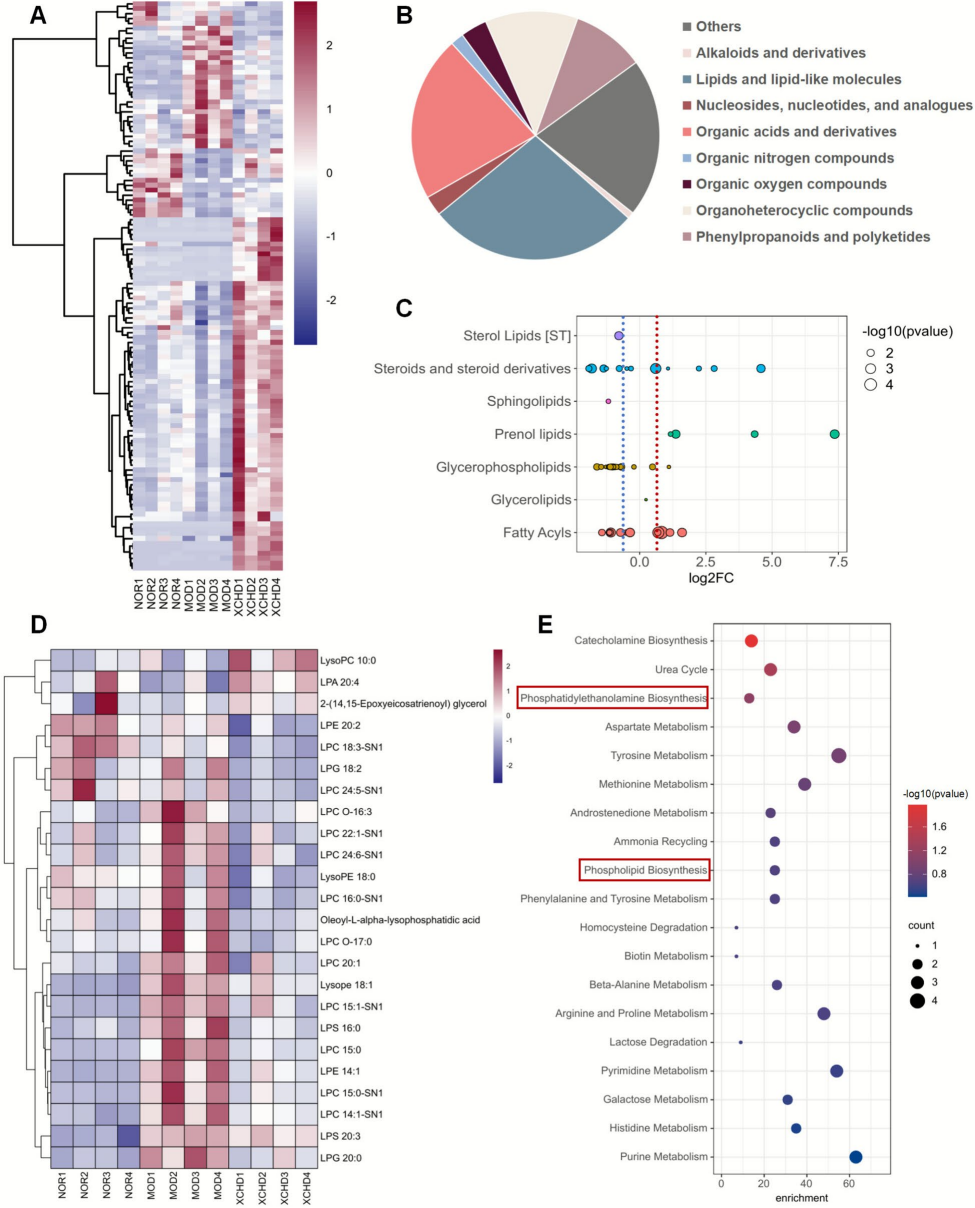
XCHD modulates HFSW-induced hepatic metabolism. Non-targeted metabolomics analysis was performed (*n* = 4). **A** Heatmap and **B** categories map of significantly differentially metabolites in the liver. **C** Bubble chart of changes in lipids and lipid-like molecules. The red line indicates log₂FC = 1. The blue line indicates log₂FC = −1. **D** Heatmap of significantly differentially expressed lipids and lipid-like molecules. **E** Pathway analysis of differential metabolites

To further investigate the role of XCHD on hepatic lipid metabolism, liver tissue non-targeted metabolomics was performed. 116 differential metabolites were detected (Fig. 3A) and classified into 9 main categories based on the Human Metabolome Database (HMDB), of which the most prominent are lipids and lipid-like molecules, followed by organic acids and derivatives (Fig. 3B). All the differential metabolites were put into MetaboAnalyst (https://www.metaboanalyst.ca/) for pathway analysis. The result suggested that XCHD regulated hepatic catecholamine biosynthesis, urea cycle, phosphatidylethanolamine biosynthesis, and so on (Fig. 3E). The subsequent stage of the investigation focused on the category of lipids and lipid-like molecules, which were divided into sterol lipids, steroids and steroid derivatives, prenol lipids, glycerophospholipids, fatty acyls, and so on. It was interesting to mention that glycerophospholipids, which are closely related to lipid droplet formation, were drastically reduced (Fig. 3C). LDs consist of neutral lipids and a surrounding monolayer of phospholipids with integral and peripheral proteins. The composition of phospholipids played a pivotal role in the process and efficiency of lipid droplet budding. Available evidences suggested an association between specific molecular geometries of lipids and their regulatory effects [23,24]. For example, diacylglycerol or phosphatidylethanolamine had been observed to exert an inhibitory effect on the budding process, while molecules characterized by opposing geometric configurations, such as lysophospholipids, promoted this dynamic event [9,25]. XCHD significantly reduced liver lysophospholipids, like LPE 20:2, LPG 18:2, LPC O-16:3, and so on, which contributed valuable insights into the mechanisms coupling lipid droplet biogenesis of XCHD in treating MAFLD (Fig. 3D).

### XCHD regulates mice liver lipid droplets formation by decreasing PLIN2 and PLIN3

**Fig. 4:**
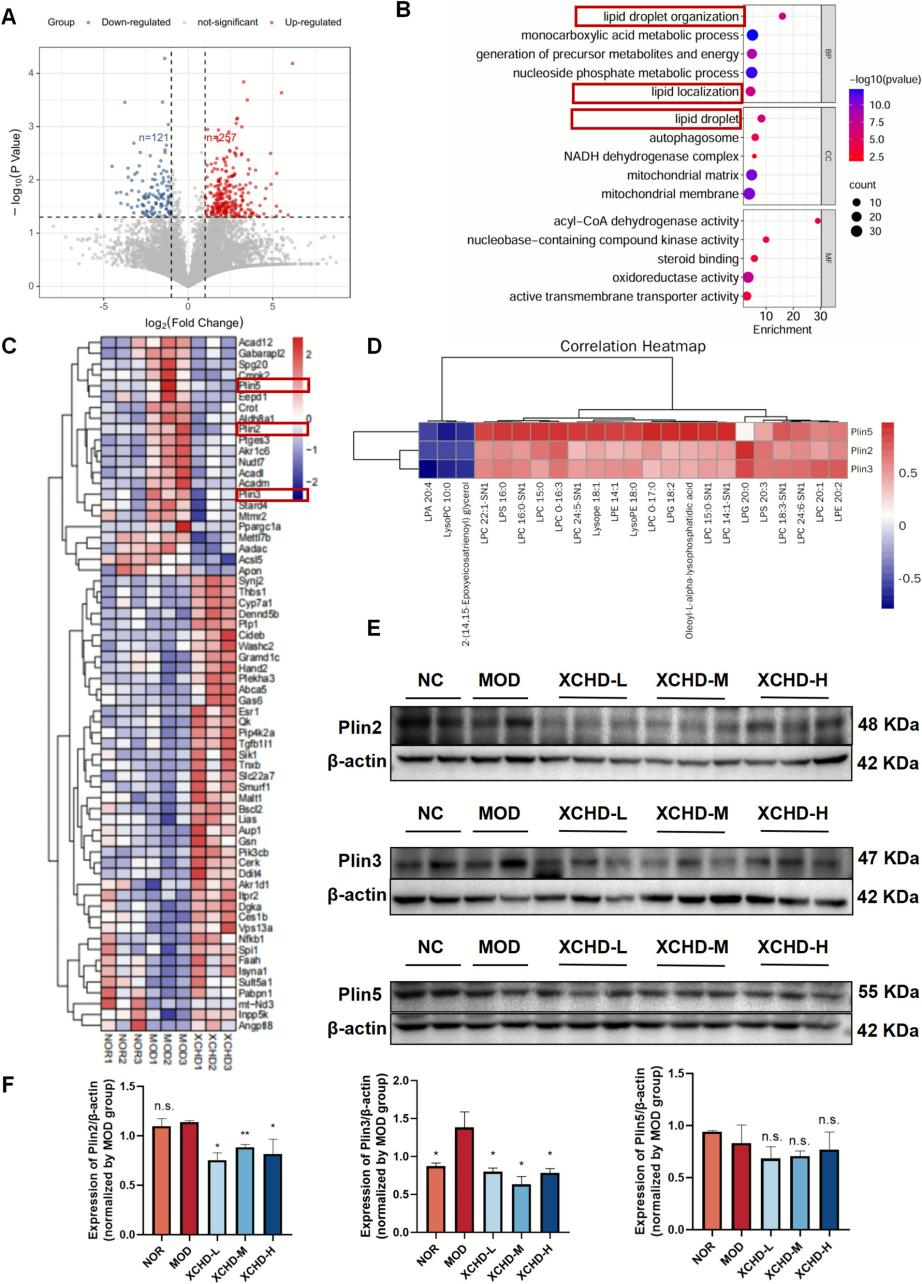
XCHD regulates HFSW-induced variations of gene expression in the liver. RNA sequencing analysis was performed (*n* = 3). **A** Volcano map of differentially expressed genes (DEGs). **B** Biological process (BP), cell component (CC), molecular function (MF) in GO enrichment of DEGs. **C** KEGG analysis of DEGs. **D** Pearson correlation analysis between PLINs and significantly differentially expressed lipids and lipid-like molecules. **E** The protein expression in liver tissue of PLIN2, PLIN3, and PLIN5 was determined by Western blot analysis. **F** The ratios of PLIN2/β-actin, PLIN3/β-actin, and PLIN5/β-actin are calculated (*n* = 3). Data are represented using at least three independent experiments as the mean ± SD. Data are represented using at least three independent experiments as the mean ± SEM. *p < 0.05, **p < 0.01, vs. MOD group

To identify the differential expression of genes in the liver regulated by XCHD in HFSW-induced NAFLD mice, three liver samples in NOR, MOD, and XCHD-M groups were selected for transcription analysis (Fig. S3A). The results showed that a total of 378 DEGs were caused by XCHD, including 257 increasing and 121 decreasing (Fig. 4A). In GO enrichment analysis, enriched DEGs were analyzed by biological process (BP), cell component (CC), and molecular function (MF), and the top 5 were shown (Fig. 4B). The results indicated that a large number of DEGs were enriched in pathways related to lipid droplet organization and lipid localization. In KEGG enrichment, a large number of metabolism-related pathways were presented, especially lipid metabolism, which was consistent with Reactome enrichment (Fig. S3B-C). On the basis of the RNA sequencing enrichment analysis, the role of XCHD on hepatic lipid metabolism in mice under the HFSW diet was further analyzed. Genes in the pathways related to fatty acid-related and lipid droplet-related pathways in GO, KEGG, and Reactome enrichment analysis were tabulated and de-emphasized. Notably, as core structural components of the LD membrane, decreased phospholipid levels analyzed by metabolomics promoted a focus on LD core protein perilipins. Both of them served as a molecular prerequisite to maintain LD structural integrity and regulate lipid storage and breakdown in liver cells. As shown in Fig. 4C, the perilipins (PLINs) family, a phosphorylated protein located on the surface of lipid droplets that has a dual regulatory function on triglyceride metabolism, both by blocking lipases approaching lipid droplets to decrease lipolysis under the basal state and by stimulating hormone-stimulated lipolysis, has demonstrated a prominent role in XCHD alleviating MAFLD. For further proof of PLINs, including PLIN2, PLIN3, and PLIN5, Pearson correlation analysis between PLINs and significantly differentially expressed lipids and lipid-like molecules was performed. PLINs are markedly correlated with lysophosphalipids highlighted by non-targeted metabolomics (Fig. 4D). As expected, the protein expression of PLIN2 and PLIN3 was significantly increased in the MOD group and almost completely reversed after the treatment of XCHD (Fig. 4E, F). However, the expression of PLIN5 was not significantly different between the MOD and XCHD groups. Consequently, it concludes that XCHD ameliorates hepatic lipid droplet formation via PLIN2 and PLIN3 in mice.

### XCHD regulates lipid droplet formation by decreasing PLIN2 and PLIN3

**Fig.5:**
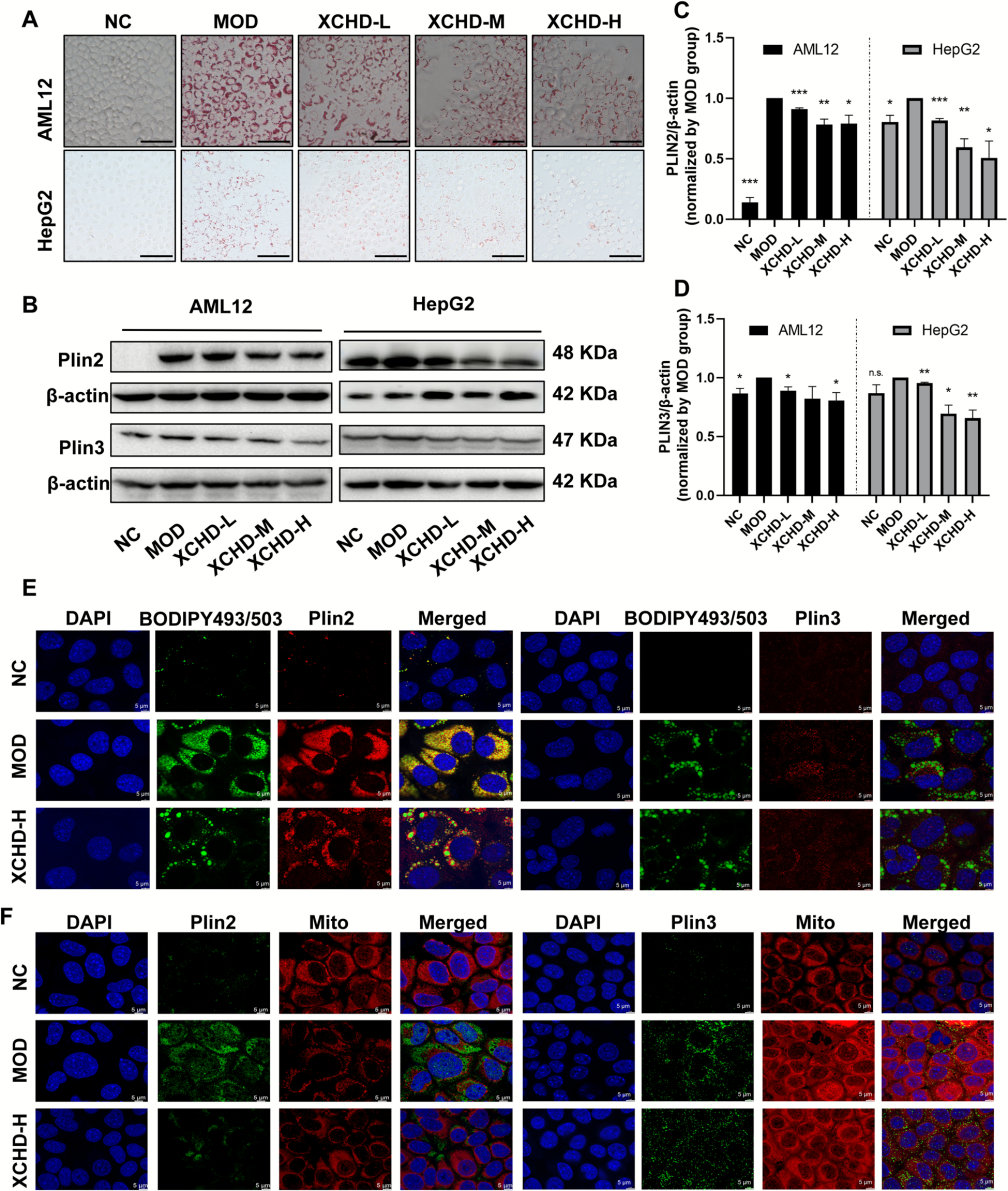
XCHD regulates HepG2 and AML12 cell lipid droplet formation. **A** Images of oil red O staining in HepG2 and AML12 cells (Scale bar = 100 μm). **B** The protein expression in HepG2 and AML12 cells of PLIN2, PLIN3 was determined by Western blot analysis. **C**, **D** The ratios of PLIN2/β-actin and PLIN3/β-actin are calculated (*n* = 3). **E** Fluorescence images of PLIN2/PLIN3 combined with BODIPY 493/503. **F** Fluorescence images of PLIN2/PLIN3 combined with Mito-Tracker Red CMXRos (Scale bar = 5 μm). Data are represented using at least three independent experiments as the mean ± SEM. *p < 0.05, **p < 0.01, vs. MOD group

To ensure the safety of drug-containing serum, a CCK8 assay was performed. The results suggested that 5%, 10%, and 20% XCHD serum showed no toxicity was observed in HepG2 and AML12 cells (Fig. S4A-B). BODIPY lipid droplet dyes can easily penetrate cell membranes and enter cells, where they bind to neutral lipids within the cells to perform specific staining. In vitro, XCHD significantly reduced lipid droplets both in HepG2 and AML12 cells, which was detected by oil red O staining (Fig. 5A) and BODIPY 493/503 (Fig. S4E-H) analysis in a dose-dependent manner. Detection of PLINs in two types of cells revealed that XCHD decreased PLIN2 and PLIN3 in both HepG2 and AML12 cells (Fig. 5B–D). Co-staining of PLINs and neutral lipids (BODIPY 493/503) revealed that, after XCHD administration, intracellular lipid droplets were significantly reduced, accompanied by a decrease in PLIN2 or PLIN3 in AML12 cells (Fig. 5E). However, for mitochondria and PLINs co-staining, the model group showed abundant large LDs; the XCHD group showed reduced LD, and residual LDs remained surrounded by mitochondria, which indicated that XCHD decreased PLINs expression in cells with highly concentrated mitochondria in the surrounding area (Fig. 5F).

### Network pharmacology

**Fig.6:**
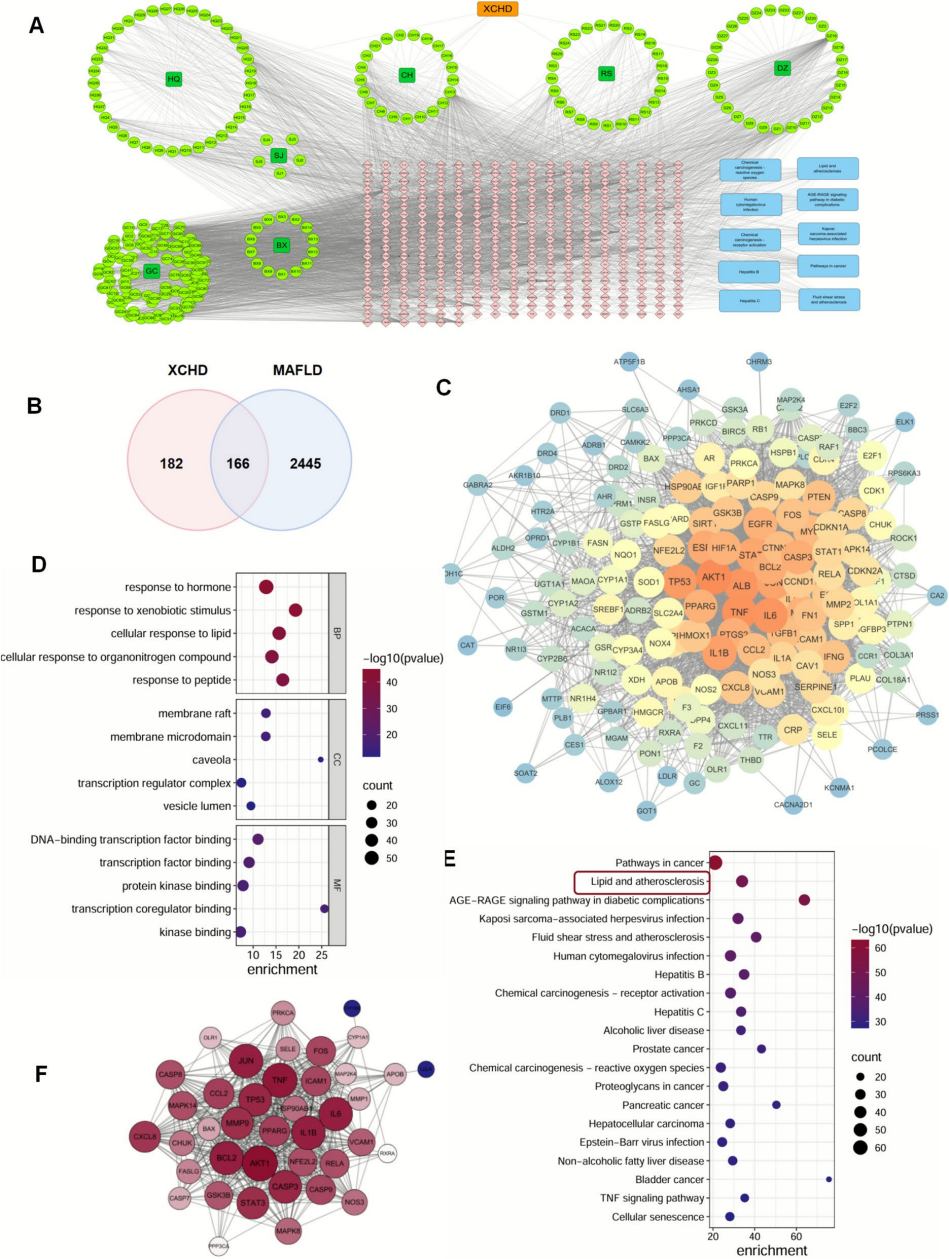
Network pharmacology analysis by XCHD and MAFLD. **A** Intersection of XCHD targets and bioactive compounds of XCHD. **B** Intersection of XCHD and MAFLD targets. **C** PPI network of genes resulting from the XCHD-MAFLD target intersection. **D** GO function enrichment analysis (including BP, CC, and MF). **E** KEGG pathway enrichment analysis. **F** PPI network of genes from the pathway about lipid and atherosclerosis

In order to find more information linked with XCHD, PLINs, and lipid droplets, Network pharmacology analysis was conducted and integrated with transcription analysis. By integrating information in the TCMSP and Swiss Target, 348 potential XCHD and targets were identified, while 2611 targets for MAFLD were identified from the CTD database (Fig. 6A, B). 166 overlapping targets were put into STRING to construct a protein–protein interaction (PPI) network (Fig. 6C) and then put into Metascape to form a GO, KEGG analysis. Result in GO was suggested that response to hormone, response to xenobiotic stimulus, cellular response to lipid would be the top 3 biological process in XCHD alleviating MAFLD (Fig. 6D). While in KEGG analysis, lipid and atherosclerosis would be the core pathway in it (Fig. 6E). After extracted genes in lipid and atherosclerosis pathway, a new PPI was established, which was prompted that genes including AKT1, BCL-2, CAPS3 and PPARG would be the core genes worked. (Fig. 6F) Compared genes with genes in the transcriptome, PPARG was also highlighted. PPARGC1B (peroxisome proliferator-activated receptor gamma coactivator 1-beta) is a coactivator of PPARs associated with diabetes mellitus and type 2 diabetes mellitus, whose important paralog of this gene is PPARGC1A. The elevation of PPARGC1B is supposed to be caused by a combination of multiple PPARs and other genes related to metabolism (Fig. 4C).

### XCHD downregulates PLIN2 and PLIN3 in a PPARs-mediated mode

**Fig.7:**
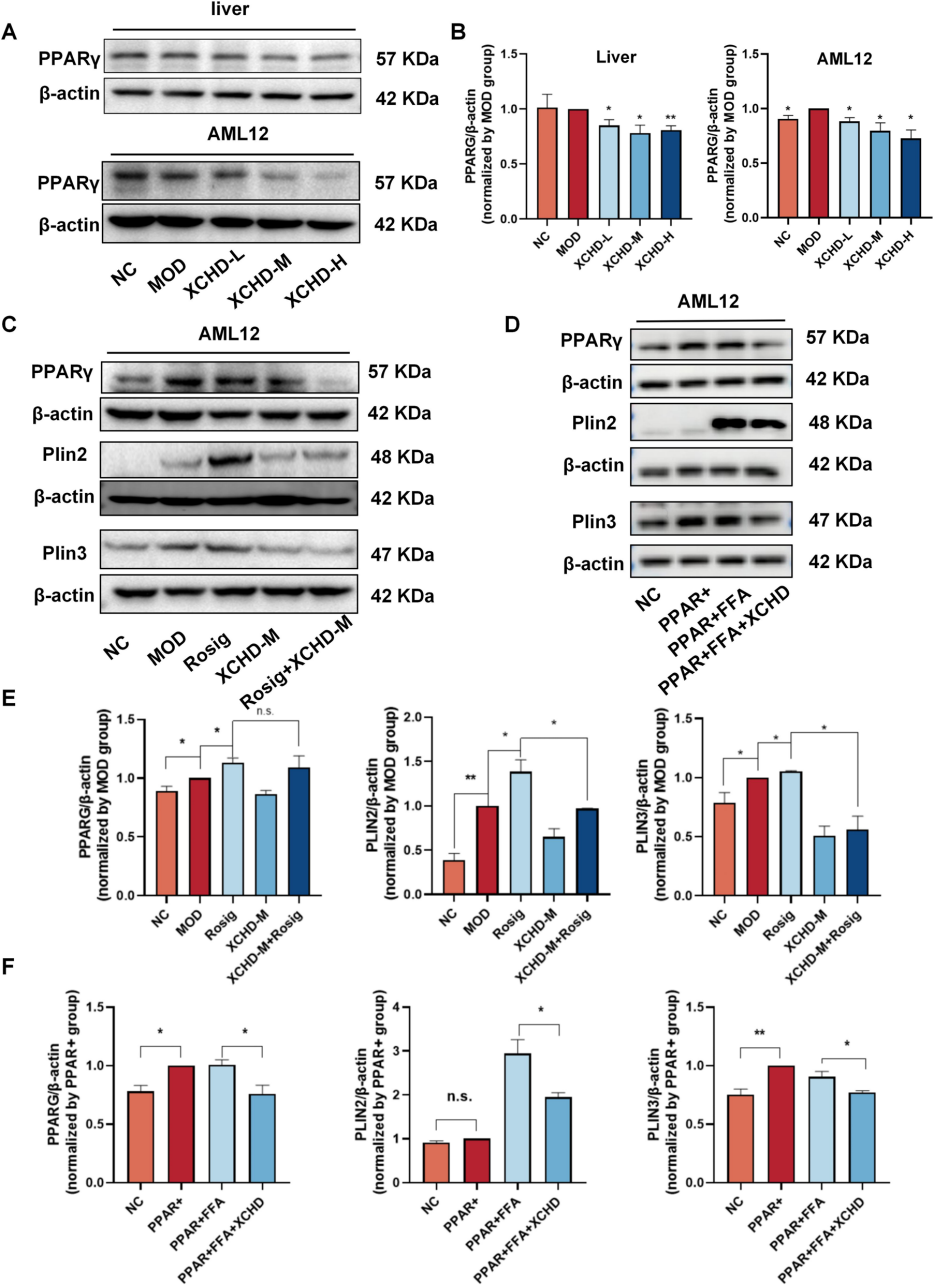
XCHD downregulates PLIN2 and PLIN3 in a PPARγ-mediated mode. **A** The protein expression in mice and AML12 cells of PPARγ was determined by Western blot analysis. **B** The ratios of PPARγ/β-actin are calculated (*n *= 3). **C** The protein expression in AML12 cells of PPARγ, PLIN2, and PLIN3 was determined by Western blot analysis after co-incubation with Rosig (a PPARγ agonist). **D** The protein expression in AML12 cells of PPARγ, PLIN2, and PLIN3 was determined after overexpression by the PPARγ vector. **E**, **F** The ratios of PPARγ/β-actin, PLIN2/β-actin, and PLIN3/β-actin by Rosig **(E)** or vector **(F)**. Data are represented using at least three independent experiments as the mean ± SD. *p < 0.05, **p < 0.01

**Fig.8:**
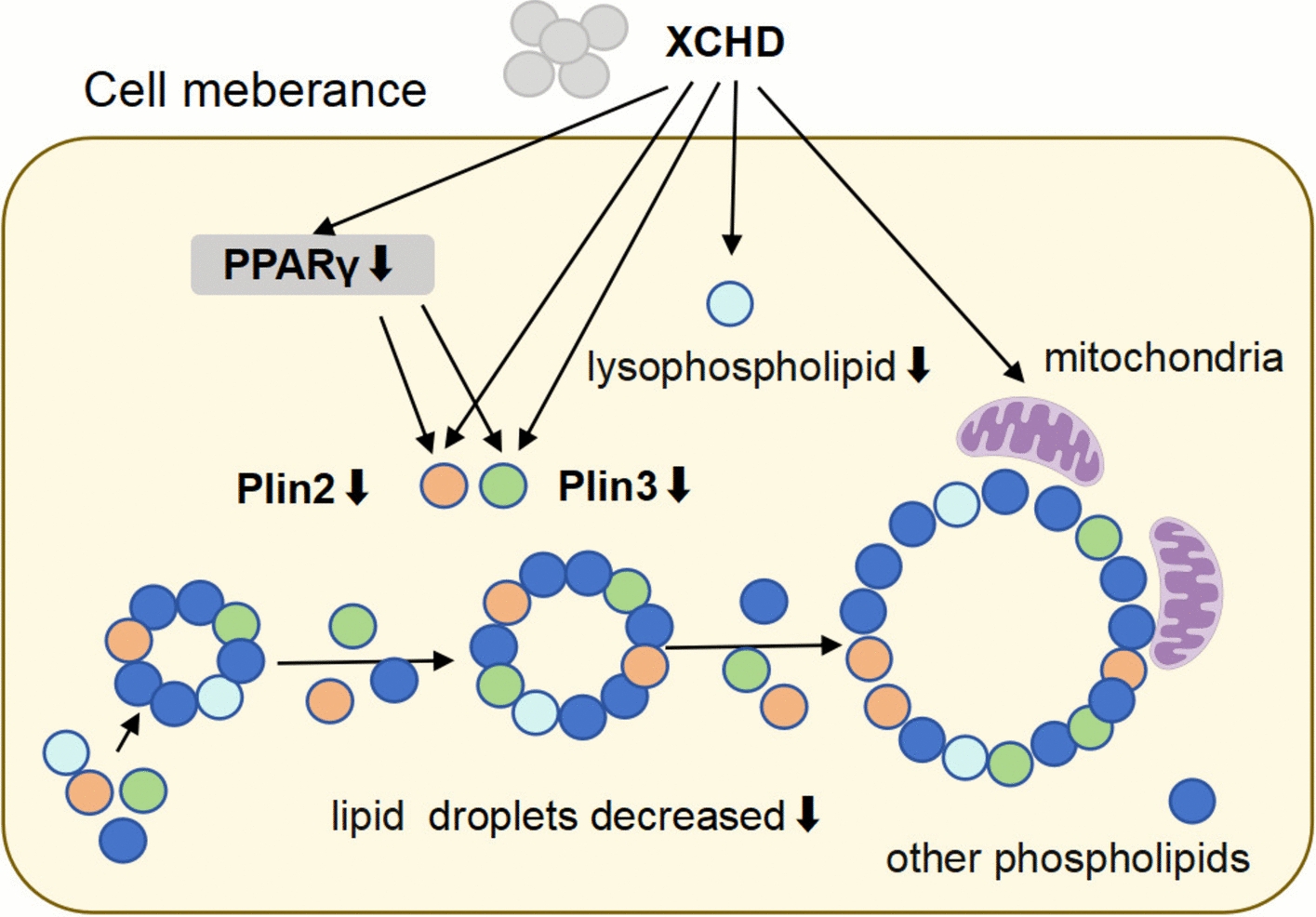
Molecular mechanism pattern diagram of XCHD in MAFLD

To investigate the role of PPARγ by XCHD in MAFLD, expression of PPARγ was detected by Western blot. The results demonstrated that XCHD significantly suppressed the expression levels of PPARγ both in vivo and in vitro (Fig. 7A, B, Fig. S5 A-B). In a systematic search for PPAR-γ target genes, it was identified perilipin was novel direct PPAR-γ target genes. To validate the direct binding of PPARγ to the promoters of PLIN2 and PLIN3, we conducted ChIP assays in AML12 cells using a specific antibody against PPARγ. The ChIP-qPCR results showed that PPARγ was significantly enriched at the PPRE regions of PLIN2 promoters, while mild for PLIN3, compared with the IgG control group (Fig. S5 I). To provide further validation of the XCHD mechanism, the inhibitory effect of XCHD was observed to be markedly attenuated by the addition of Rosiglitazone, a known PPARγ agonist and T007, a known PPARγ inhibitor. T007 significantly inhibited the expression of PLIN2 and PLIN3 in HepG2 and AML12 cells. XCHD the same function with T007 (Fig. S5 D). It is noteworthy that, while Rosig activation of PPARγ resulted in a substantial upregulation of PLIN2 and PLIN3, it did not lead to a completely elimination on inhibitory effect of PLIN2 and PLIN3 by XCHD, as evidenced by the lack of significant change in PLIN2 and PLIN3 expression between XCHD group and XCHD + Rosig group (Fig. 7C–E, Fig. S5 C). This may be related to the multi-component, multi-target synergistic action of XCHD, suggesting that its inhibition of PLIN2 and PLIN3 does not solely depend on PPARγ suppression. To further validate our hypothesis, we overexpressed PPARγ via transfection in order to rule out differences in signalling pathways by XCHD and Rosig that might be responsible for the divergent outcomes. The results of the study demonstrated that the expression of PLIN3 was significantly promoted by PPARγ expression in the absence of FFAs (in conditions that reflect the normal physiological state) (Fig. 7 D). However, no effect on PLIN2 expression was observed. Following the addition of FFA, there was a significant increase in the expression of PLIN2, as evidenced by the marked enhancement of PPARγ expression. This finding is consistent with previous reports indicating that PLIN2 upregulation is primarily driven by exogenous FFA rather than endogenously generated FFA. In accordance with prevailing expectations, XCHD led to a substantial reduction in the expression of both PLIN2 and PLIN3 (Fig. 7 D, F, Fig. S5 E). In sum, it was demonstrated that XCHD exerts regulatory effects via the PPARγ pathway to inhibit PLIN2 and PLIN3, finally reducing the lipid droplet in liver cells, while its inhibitory activity on PLIN2 and PLIN3 is not exclusively dependent on this pathway. (Fig. 8).

## Discussion

The prevailing evidence indicates that inadequate diet and lifestyle choices are the primary contributors to obesity, as well as metabolic dysfunction. These factors have been shown to be the main triggers for metabolic dysfunction-linked fatty liver disease (MAFLD). [26]. MAFLD has been regarded as the most prevalent chronic liver disorder affecting approximately 30% of the adult population, with approximately 20% of these cases classified as MASH. In order to prevent the development of MAFLD, it is imperative to emphasise the significance of controlling abnormal metabolism. However, the present treatment strategy is cumbersome and lacks effectiveness [27–29]. Over the past 20 years, traditional Chinese medicine (TCM) has garnered attention due to its efficacy in regulating metabolism and ameliorating fatty liver disease. TCM has achieved this through a multifaceted intervention, involving the inhibition of TG synthesis, the regulation of fatty acid absorption and transport, and the enhancement of lipophagy, among other mechanisms. [30]. XCHD, a classical TCM formula, is widely used in chronic liver diseases like fatty acid disease, hepatitis B, cirrhosis, and hepatocellular carcinoma [31]. Recent studies highlight that XCHD is a stellar formula in treating MAFLD, but there is a lack of research on the underlying mechanisms.

In this study, the investigation revealed that XCHD exerts a profound regulatory effect on lipid metabolism in the MAFLD model mice, operating independently of alterations in caloric intake. A substantial diminution in white adipose tissue mass is suggestive of a substantial shift in adipose lipid homeostasis, indicating either enhanced catabolism of stored lipids or suppressed adipogenic lipid synthesis within adipose depots. Concomitantly, the marked decrease in hepatic lipid content, coupled with reduced serum levels of TG, TC, and NEFA, denotes improved hepatic lipid clearance and attenuated ectopic lipid accumulation, signifying a more efficient systemic lipid processing and elimination mechanism. The parallel reductions in ALT and AST activities provide further evidence of diminished hepatocellular injury, likely secondary to the alleviation of hepatic steatosis. Moreover, improvements in insulin resistance are closely connected to the re-establishment of a healthy lipid balance, which is achieved by regulation of lipids in the blood and liver [26]. The findings, taken together, demonstrate the capacity of XCHD to modulate systemic lipid flux and have the potential in the management of MAFLD, characterised by dysregulated adiposity and hepatic lipid accumulation.

To further explore the mechanisms XCHD acts on lipid metabolism, transcriptomics and metabolomics were performed [32]. The results of the integrated GO, KEGG, and Ractome enrichment analysis of multiple omics suggest that XCHD regulates hepatic lipid droplet metabolism to alleviate MAFLD. Lipid droplets (LDs), composed of structural (phospholipids and lysophospholipids) and energy-storing lipids (triacylglycerols and free fatty acids) and surrounded by lipid protein, are pivotal to the causation of MAFLD, a disorder characterised by excessive lipid accumulation [33]. Lysophospholipids consist of long hydrophobic carbon chains and hydrophilic head groups attached to the glycerol backbone with signaling functions. During lipid droplet formation, there are localized and transient high levels of lysosomal phospholipids. Studies show that LPC stimulated LD formation from the ER in Huh7 cells [34]. Mechanistically, lysophospholipids promote LD budding by reducing ER bilayer tension and increasing the positive curvature of the LD-coating monolayer [35]. Beyond LDs' dynamics, lysophospholipids serve as intermediates in MAFLD. It is proven that LPC is elevated in NAFLD [36], correlating with disease severity, while LPE induces LDs formation in human hepatocyte-derived cell lines, underscoring their pathogenic relevance [37]. Collectively, lysophospholipids emerge as multifunctional mediators linking lipid metabolism, membrane dynamics, and signaling in LDs biology and MAFLD. XCHD significantly reduced liver lysophospholipids, like LPE 20:2, LPG 18:2, LPC O-16:3, and so on, which contributed valuable insights into the mechanisms coupling lipid droplet biogenesis of XCHD in treating MAFLD.

In particular, the surface of LDs also contains diverse proteins that are involved in lipid metabolism, membrane transport, and protein degradation. These proteins can be classified into two categories based on their location. Perilipins (PLINs) are an important class II protein localised in the cytosol and targeted to the LDs’ surface by a hydrophobic domain, including PLIN1, PLIN2, PLIN3, PLIN4, and PLN5 [38]. Through molecular biology experiments, XCHD inhibited the expression of PLIN2 and PLIN3 in vivo and in vitro to further inhibit LDs formation. PLIN2, a predominantly cytosolic protein expressed in the liver, has been implicated in the development of steatosis and insulin resistance. It adheres to the membranes of lipid droplets (LDs), restricting the access of adipose triglyceride lipase (ATGL), thereby inhibiting lipolysis. PLIN2 was overexpressed when MAFLD occurred. It was proven that specific knockout of PLIN2 would reduce mice liver triglycerides, enhance ATGL expression, lipophagy, and fatty acid oxidation by mitochondria, and mitigate LD accumulation, thereby preventing steatosis [39,40]. PLIN3, which is ubiquitously expressed, has been found to localise to new LDs, yet is displaced by PLIN2 as LDs enlarge. Consequently, PLIN3's functions remain incompletely defined. However, the reduction of PLIN3 has been shown to ameliorate steatosis and improve insulin sensitivity in models of MAFLD [41].

Numerous studies have confirmed that PLIN's function is associated with peroxisome proliferator-activated receptor (PPARs), including PPARα [42], PPARγ [43], and PPARδ [44]. The activity of hepatic PPARγ was due to the upregulation of genes involved in lipogenesis, triglyceride synthesis, and lipid droplet formation (PLINs) [45]. Accordingly, it was demonstrated that PPARγ and Retinoid X receptor (RXR) are involved in LDs formation as triggers of PLIN2 in cells [46,47]. Moreover, Numerous studies have also confirmed that PPARγ inhibition suppresses the expression of PLIN2 or PLIN3^[[48]]^. It also indicated that pharmacological inhibition of PPARγ ameliorates fatty liver [49]. Compounds from XCHD were collected by TCMSP and UPLC-MS to conduct a compound-targets network. Then, Network pharmacology was performed by interacting targets both in XHCD and MAFLD. It was suggested that PPARG would be the core gene, which was also found to have a decreasing expression in transcriptome analysis. Notably, PPARγ overexpression alone failed to induce detectable changes in PLIN2 expression under FFA-deficient conditions. This observation implied that PPARγ activation, while a known regulator of lipid metabolism-related genes, might not be sufficient to trigger PLIN2 transcription in the absence of specific lipid-derived signals. Such a finding highlights the contextual dependence of PLIN2’s regulatory network, where PPARγ’s transcriptional activity toward PLIN2 likely requires co-factors or upstream stimuli associated with lipid availability [42]. Importantly, our data align with previous research demonstrating that PLIN2 upregulation is primarily driven by exogenous FFA rather than endogenously synthesized FFA. Our study revealed that XCHD inhibited the expression of PPARγ, followed by decreased PLIN2 and PLIN3 to reduce lipid droplet formation.

Our study provides critical evidence that XCHD alleviates MAFLD by regulating liver lipid droplet metabolism, targeting the PPARγ-PLIN2/PLIN3 pathway, which provides a new perspective in MAFLD treatment. However, PPARγ was not the only factor that influenced PLIN2 and PLIN3. TCM is characterized by its multi-component nature, as its formulations or medicinal materials typically contain a variety of coexisting chemical entities, such as alkaloids, flavonoids, saponins, volatile oils, and more. These components are not present in isolation; rather, they are often responsible for the execution of their respective biological activities at different levels. However, the specific molecular mechanisms and biological processes by XCHD components through synergy and antagonism have not been fully elucidated, which remains to be explored in the future.

## Conclusions

The present study essentially demonstrated that XCHD is effective in preventing MAFLD, the potential mechanism is associated with inhibiting lipid droplet formation by decreasing lysophospholipid and lipoprotein by inhibiting PLIN2 and PLIN3. PPARγ was one of the cross in for PLIN2/PLIN3 pathway, which provides novel insights for MAFLD therapy.

## Supplementary Information

Supplementary Material 1

## Data Availability

No datasets were generated or analysed during the current study.

## Abbreviations

MAFLD: Metabolic associated fatty liver disease
XCHD: Xiaochaihu decoction
TG: Triglyceride
TC: Total cholesterol
PLIN2/Plin2: Perilipin 2
PLIN3/Plin3: Perilipin 3
PLIN5/Plin5: Perilipin 5
LDs: Lipid droplets
TCM: Traditional Chinese Medicine
CH: Bupleuri Radix
HQ: Scutellariae Radix
RS: Ginseng Radix et Rhizoma
GC: Glycyrrhizae Radix et Rhizoma
BX: Pinelliae Rhizoma
SJ: Rhizoma Zingiberis Recens
DZ: Jujubae Fructus
HFSW: contained western diet (42% kcal from fat and 0.2% cholesterol) and a high-fructose-glucose solution (18.9 g/L D-glucose and 23.1 g/L D-fructose) diet
ALT: Alanine aminotransferase
AST: Aspartate aminotransferase
NEFA: Nonesterified fatty acid
LDL-C: Low-density lipoprotein cholesterol
HMDB: Human Metabolome Database
GO: Gene Ontology
KEGG: Kyoto Encyclopedia of Genes and Genomes
PPAR: Peroxisome Proliferator-Activated Receptor
