# Supplementary material for: Unraveling the potential mechanisms of Xiaochaihu decoction alleviates metabolic associated fatty liver disease (MAFLD) by integrated transcriptomics, metabolomics, and network pharmacology
Source: Chin Med. 2026 Jan 20;21:41. doi: 10.1186/s13020-025-01310-y (PMC12817532; doi:10.1186/s13020-025-01310-y)
Supplement: Supplementary file 1 — Supplementary Material 1 [file 13020_2025_1310_MOESM1_ESM.pdf]

Supplementary figure1

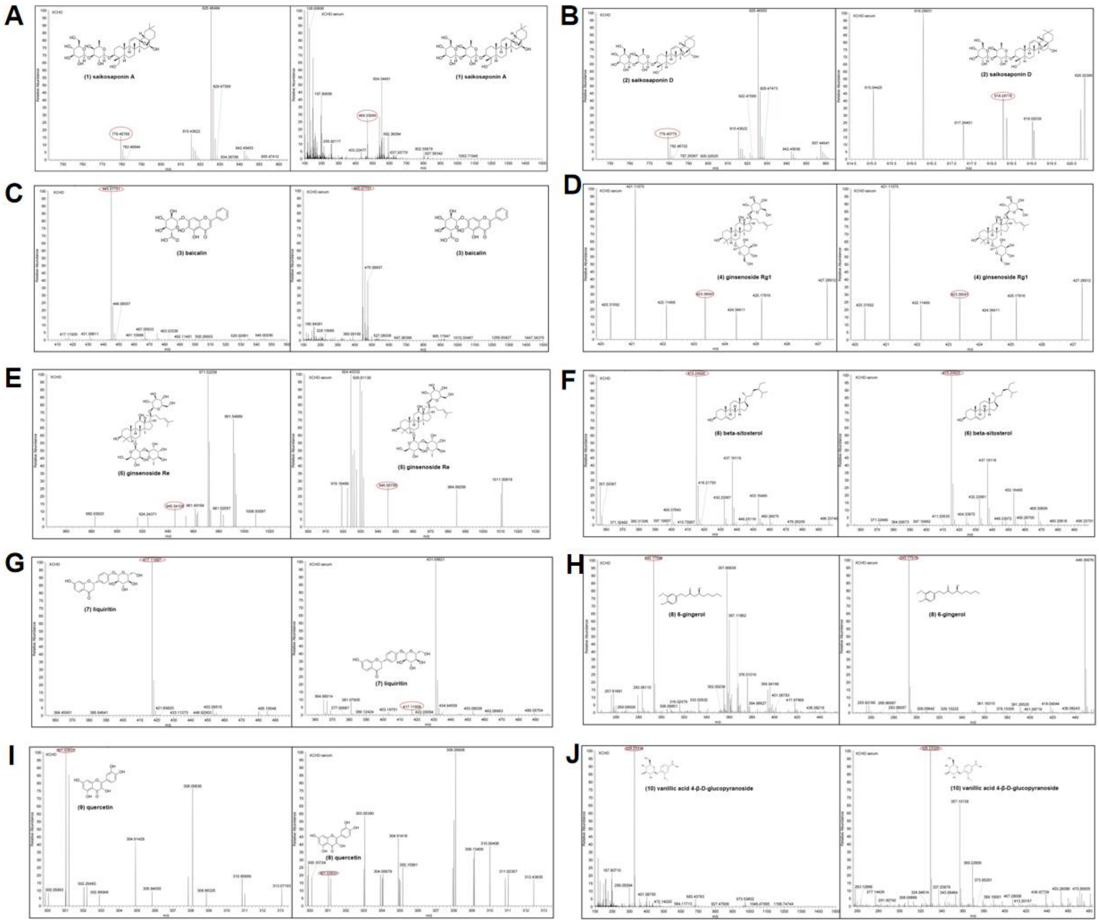

FigureS1. Detailed mass spectrum images of each components combined with their 2D structures of saikosaponin A (A), saikosaponin D (B), baicalin (C), ginsenoside Rg1 (D), ginsenoside Re (E), beta-sitosterol (F), liquiritin (G), 6-gingerol (H), quercetin (I) and vanillic acid 4-β-D-glucopyranoside (J) were shown.

Supplementary figure2

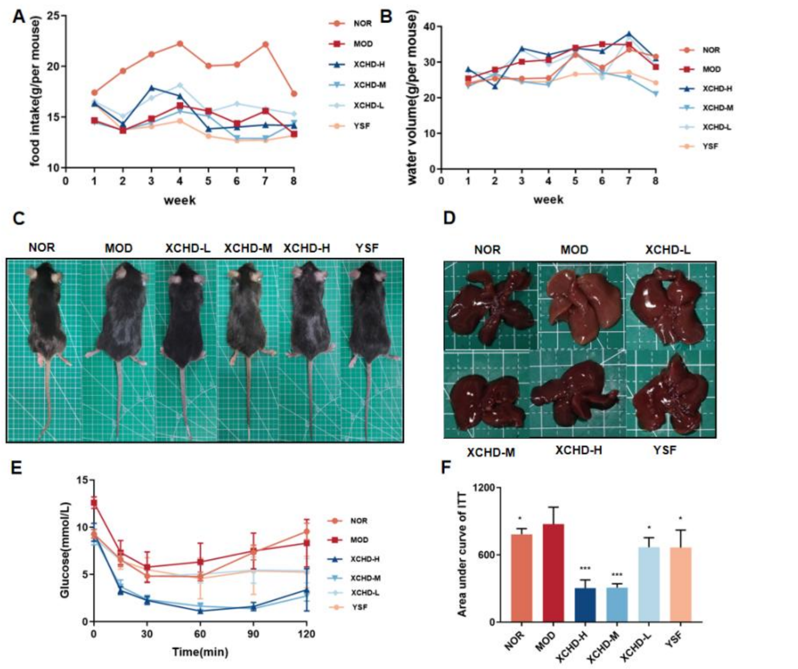

FigureS2. (A) The weekly food intake per mice. (B) The weekly water intake per mice. Typical photos of body conditions (C) and the liver (D). (E) Insulin tolerance test (ITT) of mice (n=3). (F) Area under the curve of ITT.

FigureS3.

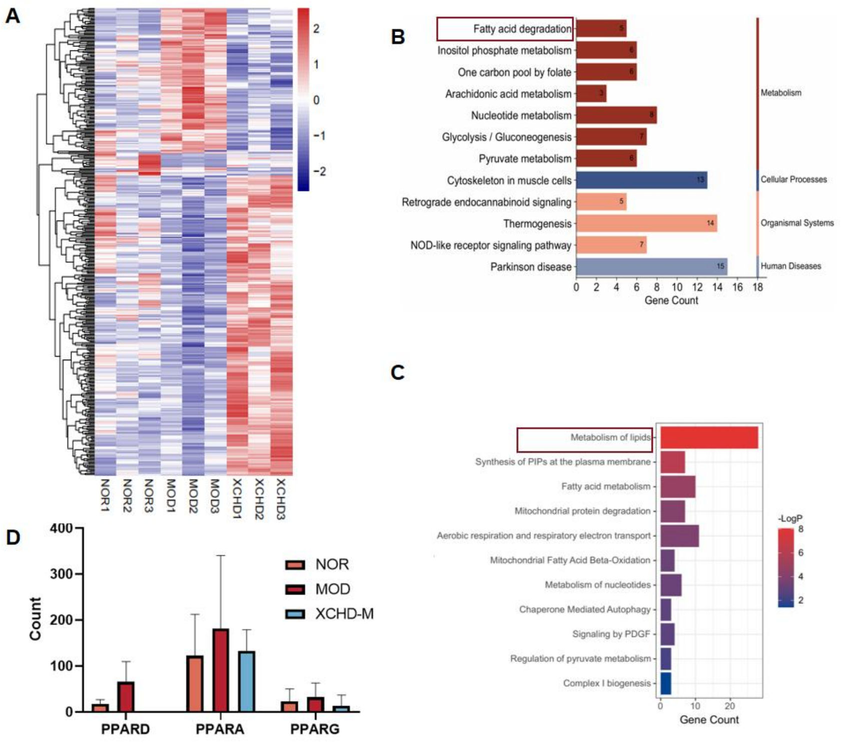

FigureS3. (A) Heatmap of significantly differentially genes in the liver. (B) KEGG analysis of DEGs. (C) Reactome enrichment analysis of DEGs. Each point in the chart represents

the enrichment level, the color from red to blue corresponds to -log10(p-value), and the size corresponds to gene count. (D) Count of PPARs by RAN-seq.

FigureS4.

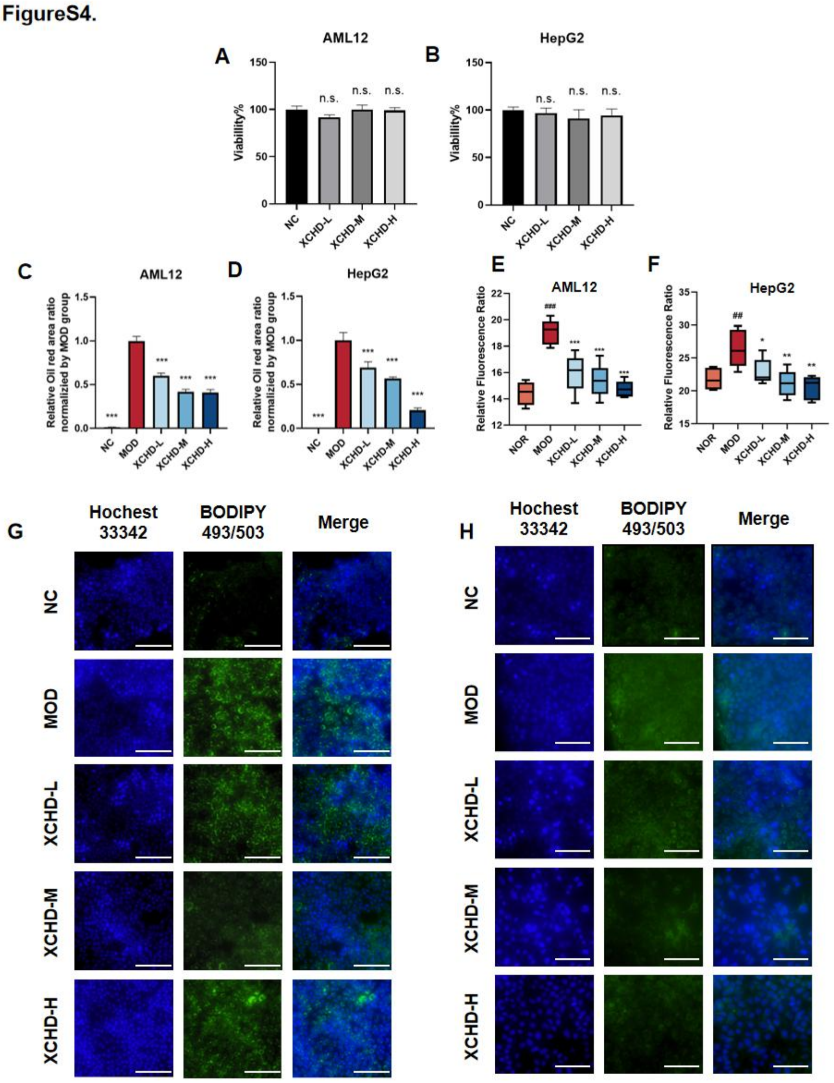

FigureS4. Cell viability% of AML12 cell (A) and HepG2 (B) cell after treated by XCHD. Relative Oil red O area ratio in AML12 cells (C) and HepG2 cells (D) normalized by MOD groups analysis by image J. Fluorescence ratio of BODIPY 493/503/Hoechst 33342 in AML12 cells (E) and HepG2 cells (F) (Scale bar= 200 μm). Images of BODIPY 493/503 staining in AML12 cells (G) and HepG2 cells (H). Data are represented using at least three independent experiments as the mean ± SD. *p <0.05, **p < 0.01 vs. MOD group

FigureS5.

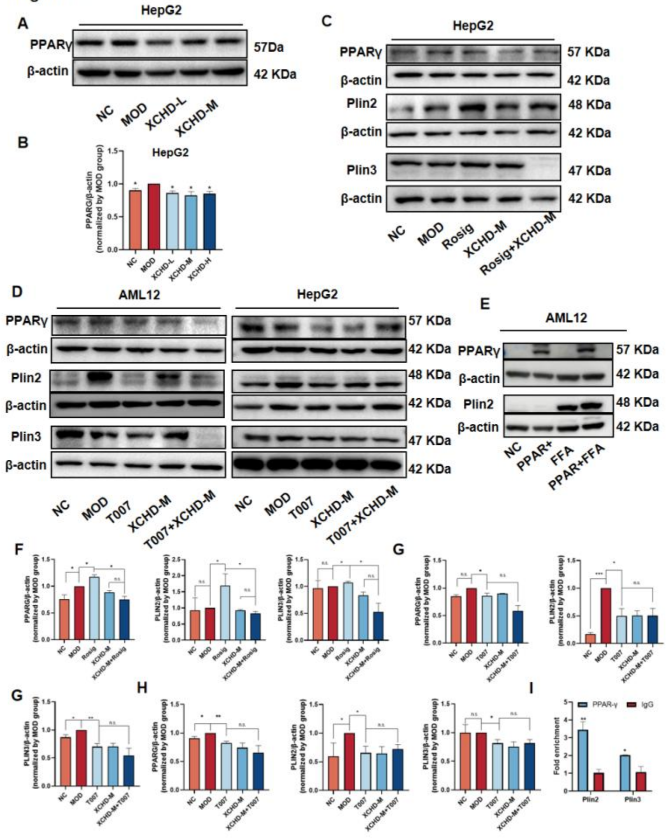

FigureS5. (A) The protein expression in HepG2 cells of PPARγ was determined by Western blot analysis. (B) The ratios of PPARγ/β-actin are calculated (n=3). (C) The protein expression in HepG2 cells of PPARγ, PLIN2, and PLIN3 were determined by Western blot analysis after co-incubation with Rosig (a PPARγ agonist). (D) The protein expression in AML12 and HepG2 cells.of PPARγ, PLIN2, and PLIN3 were determined after co-incubation with T007 (a PPARγ inhibitor). (E) AML12 cells were treated by PPARγ vector or control vector for 4h, then treated by FFA for 24h. After that, the protein expression PPARγ and PLIN2 were determined by Western blot analysis. (F) The ratios of PPARγ/β-actin, PLIN2/β-actin, PLIN3/β-actin by Rosig in HepG2 cells. The ratios of PPARγ/β-actin, PLIN2/β-actin, PLIN3/β-actin by T007 in AML12 cells (G) and HepG2 cells (H). Data are represented using at least three independent experiments as the mean ± SD. *p <0.05, **p < 0.01, XCHD vs. MOD group. (I) AML12 cells were seeded in the 10cm plates for 24 h and treated by 500 μ M FFA for 24h. Cells were taken for CHIP analysis by ChIP Assay Kit (P2078, Beyotime) and purified using DNA Purification Kit (D0033, Beyotime). In CHIP analysis, 4ul PPAR γ antibody (16643-1-AP, Proteintech) was added in the group of PPAR γ . Then qPCR were taken. PCR primers for ChIP-qPCR were: Plin2, 5'-3', TGATGTGGTCCAACCTTCGC, CTGTCCACCGCTTGTTACTCC; Plin3, 5'-3', TTGGACGACCTGAGGACTTTG, CAGAAGTTGGGTCCTTGGGTAA.
